# Functional and morphological evolution in gymnosperms: A portrait of implicated gene families

**DOI:** 10.1111/eva.12839

**Published:** 2019-07-21

**Authors:** Amanda R. De La Torre, Anthony Piot, Bobin Liu, Benjamin Wilhite, Matthew Weiss, Ilga Porth

**Affiliations:** ^1^ School of Forestry Northern Arizona University Flagstaff AZ USA; ^2^ Department of Wood and Forest Sciences Laval University Quebec City Quebec Canada; ^3^ Institute for System and Integrated Biology (IBIS) Laval University Quebec City Quebec Canada; ^4^ Centre for Forest Research (CEF) Laval University Quebec City Quebec Canada; ^5^ College of Forestry Fujian Agricultural and Forestry University Fuzhou Fujian China

**Keywords:** biotechnology, functional evolution, gene families, gymnosperms, PDR gene family, reproductive biology, stress

## Abstract

Gymnosperms diverged from their sister plant clade of flowering plants 300 Mya. Morphological and functional divergence between the two major seed plant clades involved significant changes in their reproductive biology, water‐conducting systems, secondary metabolism, stress defense mechanisms, and small RNA‐mediated epigenetic silencing. The relatively recent sequencing of several gymnosperm genomes and the development of new genomic resources have enabled whole‐genome comparisons within gymnosperms, and between angiosperms and gymnosperms. In this paper, we aim to understand how genes and gene families have contributed to the major functional and morphological differences in gymnosperms, and how this information can be used for applied breeding and biotechnology. In addition, we have analyzed the angiosperm versus gymnosperm evolution of the *pleiotropic drug resistance* (*PDR*) gene family with a wide range of functionalities in plants' interaction with their environment including defense mechanisms. Some of the genes reviewed here are newly studied members of gene families that hold potential for biotechnological applications related to commercial and pharmacological value. Some members of conifer gene families can also be exploited for their potential in phytoremediation applications.

## 1 INTRODUCTION

Gymnosperms are an ancient and widespread nonflowering plant lineage of great economic and ecological importance. With only 1,000 living species, gymnosperms represent four of the five seed plant lineages including conifers (Pinophyta), cycads (Cycadophyta), ginkgos (Ginkgophyta), and gnetophytes (Gnetophyta; Wang & Ran, 2014). Coniferous species make up 39% of the world's forests and represent great value for forestry‐dependent economies in Northern and Southern hemispheres (Armenise, Simeone, Piredda, & Schirone, 2012). Efforts to understand their biology, and genomic and functional evolution have been limited by their life‐history characteristics and large genome sizes (De La Torre et al., 2014). Recent studies highlighted the underpinnings of the major morphological, genomic, and functional differences that shaped the evolutionary divergence among gymnosperms and flowering plants.

The most noteworthy differences between angiosperms and gymnosperms certainly occur at the morphological level. Flowers, the major functional innovation in angiosperms, are assumed to have evolved through the transformation of gymnosperms' separate male and female structures into an integrated hermaphrodite structure (Niu et al., 2016; Pires & Dolan, 2012). Similarly, angiosperms developed a more efficient method of water transport through vessels, while tracheids are present in gymnosperm species (with the exception of gnetales), but also in the basal angiosperm *Amborella trichopoda*. Gene families involved in secondary metabolism such as terpene biosynthesis or various alkaloid biosynthesis pathways evolved differently in gymnosperms and flowering plants (Chen, Tholl, Bohlmann, & Pichersky, 2011; Hall, Zerbe, et al., 2013b). In this review paper, we aim to understand how a subset of well‐studied genes and gene families have contributed to the evolution of major morphological and functional differences between angiosperms and gymnosperms including their reproductive biology, water‐conducting xylem tissues, secondary metabolism and stress, and noncoding and small RNAs. In addition, we analyzed the gene family evolution of the *pleiotropic drug resistance* (*PDR*) proteins, known to play important roles in plant–environment interactions in angiosperms. Some of the gymnosperm genes reviewed here are newly studied members of gene families such as PDR that hold potential for biotechnological applications with commercial and pharmacological value. Some members of conifer gene families have potential to be exploited for improved growth on marginal or disturbed soils, by increasing the detoxification potential of spruces in phytoremediation applications.

## 2 GENOMIC EVOLUTIONARY DIFFERENCES BETWEEN ANGIOSPERMS AND GYMNOSPERMS

Before the extensive radiation of flowering plants during the late Cretaceous, gymnosperms dominated the world flora for almost 200 million years (Pennisi, 2009; Pires & Dolan, 2012). Extreme climatic shifts over the Cenozoic resulted in major extinctions in the gymnosperm lineage, which may account for the low diversity of extant gymnosperms in comparison with their sister seed plant clade (Crisp & Cook, 2011; Leslie et al., 2012). Extinctions were more pronounced in the Northern hemisphere in which older lineages were replaced by those better adapted to cooler and drier environmental conditions, resulting in higher species turnover rates in Pinaceae and Cupressaceae, compared to southern lineages (Leslie et al., 2012). More recently, climatic changes during the last Glaciation strongly shaped species distributions and patterns of speciation and adaptation for many Northern hemisphere gymnosperms which went through cycles of contraction and expansion from refugia (Shafer, Cullingham, Côté, & Coltman, 2010).

While angiosperm evolution has been shaped by whole‐genome duplication (WGD) events leading to higher speciation rates and the development of key functional innovations, gymnosperm genomes have been less dynamic (Landis et al., 2018; Soltis & Soltis, 2016; Vanneste, Maere, & Van de Peer, 2014). The rarity of WGD, paucity of chromosomal rearrangements, and slow mutation rates have led to low levels of structural genomic and morphological variation among species, and low speciation rates in gymnosperms (De La Torre et al., 2014; De La Torre, Li, Van de Peer, & Ingvarsson, 2017; Jaramillo‐Correa, Verdu, & Gonzalez‐Martinez, 2010; Leitch & Leitch, 2012; Pavy et al., 2012). In the presence of polyploidy and retro‐transposition, angiosperms have developed mechanisms to counteract the increase in genomic DNA by replication or recombination‐based errors generating indels, and unequal recombination between sister chromosomes (Grover & Wendel, 2010; Leitch & Leitch, 2012).

Although polyploidy is largely absent in gymnosperms (exceptions are *Sequoia*, *Pseudolarix*, and *Ephedra*), a combination of a massive accumulation of long‐terminal repeat retrotransposons (LTR‐RTs), together with limited removal of transposable elements through unequal recombination, has resulted in very large genome sizes (mean 1C = 18.8 pg; De La Torre et al., 2014; Leitch & Leitch, 2012; Nystedt et al., 2013). Recent studies revealed transposable elements make up to 74%, 76.58%, 79%, and 85.9% of the genomes of *Pinus taeda*, *Ginkgo biloba*, *Pinus lambertiana*, and *Gnetum montanum*, respectively (Guan et al., 2016; Neale, Martínez‐García, Torre, Montanari, & Wei, 2017; Neale et al., 2014; Wan et al., 2018; Wegrzyn et al., 2014). A comparative analysis among six diverged gymnosperms suggested the diversity and abundance of transposable elements is widely conserved among gymnosperm taxa (Nystedt et al., 2013). However, a more recent study focused on gnetophytes (*Gnetum*, *Welwitschia*, *Ephedra*) suggests higher frequencies of LTR‐RT elimination due to recombination‐based processes of genome downsizing may explain the smaller sizes of gnetophytes in comparison with other gymnosperm genomes (Wan et al., 2018).

Despite significant variations in noncoding regions of angiosperm and gymnosperm genomes, both plant lineages have comparable numbers of genes and gene families. Sequence similarities of expressed genes are 58%–61% between conifers and angiosperms, and 80% within Pinaceae (Prunier, Verta, & MacKay, 2016; Rigault et al., 2011). This suggests that functional differences observed between seed plant lineages may have evolved as a consequence of differences in rates of nucleotide substitution, frequency of copy number variant (CNV) formation (Prunier, Caron, & MacKay, 2017; Prunier, Caron, Lamothe, et al., 2017; for a discussion of poplar vs. spruce CNVs see Prunier et al., 2019), and/or differential gene family expansion or contraction (Zhou et al., 2019). A recent analysis of protein‐coding genes across a broad phylogeny suggested slower rates of molecular evolution (number of synonymous substitutions dS and mutation rates), but higher substitution rate ratios (dN/dS) in gymnosperms than in angiosperms (De La Torre et al., 2017). Higher levels of dN/dS in gymnosperms suggest stronger and more effective selection pressures probably due to larger effective population sizes, especially in the Pinaceae (De La Torre et al., 2017). In addition, gymnosperms generally present high levels of within‐population genetic diversity, while long‐distance gene flow of wind‐dispersed pollen between highly outcrossed populations leads to rapid decay of linkage disequilibrium and low among‐population genetic diversity (De La Torre et al., 2014, 2017; Porth & El‐Kassaby, 2014). Higher gene turnover, which probably explains a higher species turnover, has been observed in Pinaceae. Although the cause of this is unknown, it is being suggested that this trend might be explained by an elevated frequency of gene CNVs, although rates of CNV formation in Pinaceae or any other gymnosperms are unknown (Casola & Koralewski, 2018).

**Figure 1:**
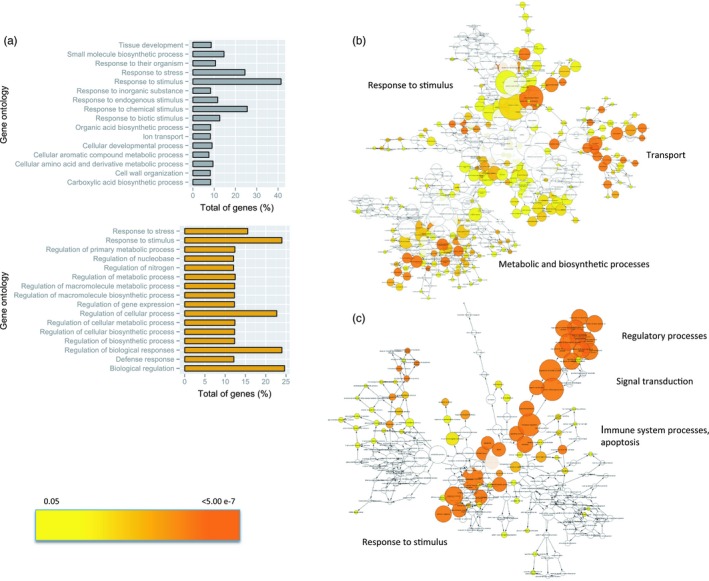
Functional enrichment of genes in gene families showing differential expansions between gymnosperms (*Picea abies*) and angiosperms (*A. thaliana*). (a) Barplot showing significant (*p* < 0.0001) enriched gene ontologies for gymnosperms (gray bars) and angiosperms (yellow bars); (b) Gene interaction network showing significant gene ontologies for gymnosperms species; (c) Gene interaction network showing significant gene ontologies for angiosperms species. *p*‐Values vary from 0.05 (yellow) to <5.00e‐7 (dark orange) according to the color scale in left bottom (A. De La Torre & P.K. Ingvarsson, unpublished). *P. abies* genome version 1.0 (Nystedt et al., 2013; http://congenie.org) was used for this analysis

Because gymnosperms predate angiosperms, most differential gene family expansions between angiosperms and gymnosperms seem to have occurred either by loss of genes in angiosperms (most likely scenario) or gain in gymnosperms (by neofunctionalization or subfunctionalization). Large expanded paralogous gene families such as leucine‐rich repeats, cytochrome P450, MYB, and others (Table 1) have been observed in gymnosperms (De La Torre, Lin, Van de Peer, & Ingvarsson, 2015; Neale et al., 2014; Pavy et al., 2013; Porth, Hamberger, White, & Ritland, 2011; Warren et al., 2015). While comparing differentially expanded gene families using whole‐genome data, our study found that *Picea abies*' larger gene ontologies, compared to those of *Arabidopsis thaliana*, are the consequence of the species' ability to respond to diverse stimuli (biotic and abiotic stress), transport mechanisms, and a variety of specific metabolic and biosynthetic processes (Figure 1).

**Table 1:** Gene families showing differential expansions in gymnosperms

Gene family	Function	Taxon	Reference
MYB	Defense response, vascular development	*Pinus taeda, Picea glauca*	Bedon et al. (2010); Patzlaff et al. (2003)
NB‐LRR	Disease resistance	*Picea abies*	Fossdal et al. (2012)
sRNA's (SGS3, DCL1)	Epigenetics, transposable element silencing	*P. taeda, Pinus lambertiana, P. abies*	Gonzalez‐Ibeas et al. (2016); Yakovlev, Carneros, Lee, Olsen, and Fossdal (2016)
Phenylalanine ammonia lyase (PAL)	Lignin biosynthesis	*P. taeda*	Bagal, Leebens‐Mack, Lorenz, and Dean (2012)
Ty3/Gypsy	Transposable element	*P. abies, Pinus palustrus, Abies concolor, Podocarpus totara* and others	Nystedt et al. (2013); Friesen, Brandes, and Heslop‐Harrison (2001)
Cytochrome P450	Monoterpenoid production	*Thuja plicata*	Gesell et al. (2015);
		*P. glauca*	Warren et al. (2015)
miR390‐TAS3‐ARF	Auxin signaling	*P. abies*	Xia, Xu, Arikit, and Meyers (2015)
CslE/J/G‐like	Cellulose synthesis	*P. abies, Cryptomeria japonica, Pinus banksiana* and others	Yin et al. (2014)
Dehydrins	Drought, cold tolerance	*P. glauca, Abies balsamea, Larix laricina* and others	Stival Sena et al. (2018); Rigault et al. (2011)
Glucosinolate biosynthesis and α‐bisabolene synthase	Terpene‐mediated defense	*Ginkgo biloba*	Guan et al. (2016)
FSL2 and EFR	Bacterial infection defense	*G. biloba*	Guan et al. (2016)

Sequence variation leading to neofunctionalization in large paralogous families might have resulted in the evolution of lineage‐specific expansions between angiosperms and gymnosperms or within gymnosperms lineages (e.g., *Picea* and *Pinus*; Gonzalez‐Ibeas et al., 2016). Conifer‐specific gene families have been observed in the Terpene synthase (TPS) subfamily (Hall, Zerbe, et al., 2013b; Shalev et al., 2018; Warren et al., 2015), in specific subfamilies of P450s such as the CYP720Bs subfamily (Geisler, Jensen, Yuen, Madilao, & Bohlmann, 2016; Warren et al., 2015) or the CYP750 family (Gesell et al., 2015; Porth et al., 2011), and in transcription factors such as mTERF involved in abiotic stress and plant growth (Gonzalez‐Ibeas et al., 2016). F‐box proteins that are subunits of the E3 ubiquitin ligase aggregations also known as the SCF quaternary complex (SKP1, Cullin1, F‐box protein, and Rbx1) also reveal conifer‐specific gene families (Gonzalez‐Ibeas et al., 2016).

## 3 REPRODUCTIVE BIOLOGY

The reproductive biology in gymnosperms is characterized by a largely outcrossing mating system, predominant anemophily (wind pollination) and wind‐mediated seed dispersal. Other characteristics that differ between gymnosperms and angiosperms are the presence of uncovered seeds (lack of fruit), a *haploid* nourishing tissue (megagametophyte) surrounding the diploid embryo in the developing seed, and temporary polyembryony.

Genomic research on the reproductive biology of gymnosperms is not as advanced as in angiosperms (Cairney & Pullman, 2007; Rodrigues, De Vega, & Miguel, 2018). This is not surprising given the lack of genetic mutant lines required for discovering genes involved in plant development and reproduction (these resources are largely available for model plant species such as *A. thaliana*: the SALK lines, e.g.). Knowledge about the genetic underpinnings of reproductive and seed development is scarce in conifers, with *Pinus* being the most studied conifer genus to date, and *Picea* to a lesser extent. Recent studies in *Picea* suggest individual or multiple gene family members involved in reproductive development have gene expression‐based relationships with tree growth and environmental stress (abiotic, biotic defenses); however, the exact molecular nature of their involvement is unknown (Porth et al., 2012; Porth, White, Jaquish, & Ritland, 2018).

Some of the most thorough reproductive development studies on pines have been on trees growing under somewhat less natural conditions and therefore prone to artificial stress (such as botanical garden settings). This has resulted in a higher frequency of anomalies such as bisexual reproductive structures (hermaphroditism) than it is expected under natural conditions (Flores‐Renteria et al., 2011; Niu et al., 2016). Nevertheless, teratology and hermaphroditism might have been common in early and now extinct gymnosperms (Rudall, Hilton, Vergara‐Silva, & Bateman, 2011). While species members of the Pinaceae family are mostly monoecious (gymnosperms overall are mostly dioecious), their male and female reproductive structures are unisexual, and megasporophylls and microsporophylls occupy distinct locations on a tree's shoot. The female structures develop at the top while male structures form at the base of a shoot in normal tree development (Niu et al., 2016), making self‐pollination difficult, and thus helping to maintain outcrossing rates and genetic diversity (Porth & El‐Kassaby, 2014). Furthermore, incompatibility mechanisms (pollen discrimination) in conifers occur within the ovule and are mostly postzygotic (Owens, Takaso, & Runions, 1998).

In order to facilitate pollen release and dispersal through wind travel, gymnosperms' male reproductive structures (male cones; pollen grains) have evolved an impressive diversity of male cone positioning, and grain shapes (Lu et al., 2011). This is seen as a necessity to overcome the innate constraints from gymnosperm's heavy (but not always exclusive) reliance on anemophily. In contrast, angiosperms evolved flowers with attractive colors and fragrances as signals for pollination by insects and other animals, pollen and nectar rewards as food source for the pollinator, as well as fruits for their seeds' protection but also dispersal by animals. Ovular secretion is also crucial to reproduction in gymnosperms as it fosters pollen germination and pollen tube growth, eventually leading to fertilization of an egg cell within gymnosperms' archegonia. Yet, virtually nothing is known about the molecular genetic basis of ovular secretion, an important characteristic in gymnosperms' megagametophytes (Zhang & Zheng, 2016). A recent study on thirteen species representing all five main lineages of extant gymnosperms (Nepi et al., 2017) suggested that oval secretion chemical profiles showing higher levels of carbohydrates and certain amino acids can identify recent or hint at ancestral gymnosperm populations that are or were insect‐pollinated, respectively. Pollination drops functioning as gymnosperm nectar have not been chemically analyzed (Von Aderkas, Prior, & Little, 2018), precluding a direct angiosperm–gymnosperm comparison at the moment. However, both gymnosperm ovular secretions and angiosperm floral nectaries have shown selection over time driven by insects and their nutritive needs related to a higher energy metabolism required for flight (Nepi et al., 2017), suggesting a similar adaptive mechanism. In any case, important insect pollinator–plant host shifts took place during angiosperm radiation in mid‐Cretaceous, leaving few survivors of previously common gymnosperm–insect pollinators either because of extinctions or insects' transition to angiosperms' plant hosts (Peris et al., 2017). Anemophily could have also been an ancient event in angiosperms due to its importance in pioneer habitats (Gottsberger, 1988).

Archegonia develop from initial cells within the female gametophyte of the ovule through subsequent rounds of divisions giving rise to (outward) neck cells, and the central cell. This later develops into the large egg cell and the small ventral canal cell, which degenerates as the egg cell matures. Despite its importance, the molecular regulation of archegonia development in the ovule of cone‐bearing gymnosperms has not been extensively studied, and the role of archegonia in egg fertilization is largely unknown (Zhang & Zheng, 2016). The major challenge for such studies is the long duration of the pollination process (up to 13 months for pines) compared to the short period of time required for zygote formation. Archegonia were not found to produce pollen‐specific signals, but neck cells might produce these (Zhang & Zheng, 2016). Evidence from lower archegoniatae such as ferns and bryophytes suggests auxin‐responsive genes might be involved in reproductive organ morphogenesis, differentiation, and cell turnover related to archegonial development (Zhang & Zheng, 2016). Some evidence also hints at a role for the *arabinogalactan protein* (*AGP*) gene family. Glycosylated AGPs could be involved in egg cell–sperm recognition, and their expression within the nucellus cells of the mature egg cell may be involved in promoting the targeted pollen tube growth (AGPs' glycosylation‐level dependent; Zhang & Zheng, 2016). *WUSCHEL‐related homeobox* (*WOX*) transcription factor genes are implicated in conifer embryo formation and differentiation (patterning) which was tightly linked to polar auxin transport (*ibidem*).

As stated earlier, most genomic resources aimed at in‐depth elucidation of the molecular basis of conifers' reproductive developmental stages were developed for *Pinus* (*Pinus tabuliformis*: Du et al., 2017; Niu, Yuan, Zhang, Chen, & Li, 2014; Niu et al., 2015; Niu et al., 2016; *Pinus sylvestris*: Avia, Kärkkäinen, Lagercrantz, & Savolainen, 2014); *Picea* (Carlsbecker et al., 2013; Vázquez‐Lobo et al., 2007); and recently for *Cryptomeria japonica* (Katahata, Futamura, Igasaki, & Shinohara, 2014; Tsubomura, Kurita, & Watanabe, 2016). The flower development gene families (*MADS‐BOX*, *LFY/NDLY*, phosphatidylethanolamine‐binding protein (PEBP) *FLOWERING LOCUS T/TERMINAL FLOWER1*‐like genes) are ancient, estimated to have been present before 300 Mya, predating the divergence of seed plants (Liu, Xiong, Li, & Fei, 2018; Moyroud et al., 2017). The first evidence of the importance of *MADS‐box* genes for conifer reproductive organs dates back to 2002 (reviewed in Zhang & Zheng, 2016). The *bona fide* *FT* locus is absent from gymnosperms; therefore, it represents an innovation in the angiosperm lineage (Klintenäs, Pin, Benlloch, Ingvarsson, & Nilsson, 2012). However, cryptic *FT* gene homologs in gymnosperms were identified through phylogenetic reconstruction in three monophyletic clades (*FT‐like*, *TFL1‐like* and *MOTHER OF FT AND TFL1‐like* PEBP genes). None of these cryptic *FT* genes induce flowering (Liu et al., 2018). In gymnosperms, the *TFL1‐like* gene shows predominant expression in cone development of both sexes. For spruce, *FTL1* is expressed in male cone development and *FTL2* in female cone development. In addition, gymnosperm‐derived *FTL2* genes are implicated in growth rhythm regulation (Liu et al., 2018). Recent evidence shows an important link between *FT*‐*like* and *MADS‐box* gene families, and growth rhythm control, bud set and growth cessation (*FT‐like*: e.g., *P. sylvestris*: Avia et al., 2014; *P. abies*: Karlgren, Gyllenstrand, Clapham, & Lagercrantz, 2013; *MADS‐box*: Petterle, Karlberg, & Bhalerao, 2013). This suggests early molecular actors in reproductive development and seasonal growth cessation are similar.

Investigating aberrant reproductive structures for the analyses of gender expression in cones can provide important insights into the complete or partial transformation of male cones into female cones (homeosis). Of particular consideration is the molecular study on teratological reproductive structures. Niu et al. (2016) demonstrated that both male and female structures of bisexual cones were functional in *P. tabuliformis* (high pollen grain germination rate, seed producing conelets); however, the transcriptomes of the male structures from the bisexual cones were found to be distinct. The authors identified key regulators among *MADS‐box* genes (i.e., *PtDAL1*, *PtDAL2*, *PtDAL3*, *PtDAL4*, *PtDAL10*, *PtDAL14*, *PtMADS1* and *PtMADS3*), as well as the two *LFY/NDLY* genes (the latter usually only expressed in female cones) that may have resulted in the presence of female structures in a male cone. Therefore, the observed sex transformation was the result of stark shifts in their gene expression patterns. Because male‐to‐female transformation in these bisexual cones was incomplete, the male‐to‐female homeosis did not involve a reduction in *PtDAL11* and *PtDAL13* expression, providing support that these two genes are essential to maintain the ancestral function in sex determination (male cone) and specify the pollen‐bearing organs generated by this meristem (Niu et al., 2016). Another seed cone developmental mutant termed *P. abies acrocona*, characterized by early cone setting and homeotic transformations of vegetative shoots into female cones, revealed that gymnosperm *LFY/NDLY* genes are involved in reproductive initiation. This observation was based on the exceptionally high *PaNDLY* expression in the axis of the *acrocona* shoot at the transition from vegetative to female identity (Carlsbecker et al., 2013).

Finally, a single fertilization event within the ovule produces a diploid embryo that develops within a haploid female gametophyte. At early seed development, polyembryony is also an important reproductive feature in conifers, whereby multiple archegonia can be fertilized by different pollen grains. In all cases though, only the dominant embryo persists and matures while all others are aborted. The molecular basis of embryo persistence is unknown (Cairney & Pullman, 2007). The embryo suspensor stage is a critical stage in early embryonic development as it helps the embryo to grow within the female gametophyte, and to benefit from nutrient absorption while it enlarges. Gymnosperms contain genes of very similar sequence to angiosperm embryogenesis‐regulating genes (Cairney & Pullman, 2007). The comparative synthesis by Cairney and Pullman revealed that in gymnosperm embryogenesis, subtle molecular interactions, spatially and temporally controlled gene expression, and few unique regulatory proteins can achieve differences in embryonic structure and development. One important example is the above‐mentioned *WOX* transcription factor genes.

Recently, transcriptomic studies on embryogenesis in pines (*P. sylvestris*; *Pinus pinaster*; *P. lambertiana*; *Araucaria angustifolia*) and spruces (*P. abies*; *Picea balfouriana*) have been published (reviewed in Rodrigues et al., 2018). Nevertheless, in order to better understand gymnosperms' unique regulatory networks, any functional analysis of conifer developmental genes must be conducted by expressing these genes in a conifer. Therefore, the development of a robust, easy‐to‐use and broadly applicable transformation system for conifers constitutes a prerequisite to a better understanding of several aspects of this phylum's cell and molecular biology (Cairney & Pullman, 2007). Up until now, this has not been achieved. More recent studies revealed a crucial role for small noncoding RNAs, and some of their target genes were revealed for the regulation of seed development and in embryo development (Rodrigues & Miguel, 2017). Niu et al. (2015) identified such sRNAs specifically for male and female cones of *P. tabuliformis*, and with higher activities in the female than in the male reproductive structures. The miR156‐SPLs, miR159‐MYBs, miR172‐ AP2Ls, miR319‐TCP, and miR396‐GRFs interacting pairs found for this pine species coincided with those in angiosperms' reproductive development, suggesting ancient evolutionary histories of these sRNA regulatory pathways (Niu et al., 2015).

## 4 WATER‐CONDUCTING XYLEM TISSUES

### 4.1 Cellulose/hemicelluloses synthases and their regulation

Cellulose, composed by a (1 → 4)‐β‐d‐glucan chain, is one of the most important components of the plant cell wall and is also a decisive factor controlling plant cell mechanical properties (Kumar, Atanassov, & Turner, 2017; Sarkar, Bosneaga, & Auer, 2009). The evolution of cellulose enhanced plant cell adaptation ability to respond to changing environments (Sarkar et al., 2009). Cellulose is biosynthesized by cellulose synthases (CesA) at the plasma membrane (Meents, Watanabe, & Samuels, 2018). The *CesA* family belongs to the *CesA* superfamily including *CesA*‐*like* (*Csl*) gene families which harbor nine *Csl* subfamilies (*Csl*A/B/C/D/E/F/G/H/J) (Yin, Johns, Cao, & Rupani, 2014). Csl subfamily proteins catalyze the biosynthesis of hemicelluloses, another polysaccharide component in the plant cell wall with a backbone similar to cellulose (Cosgrove, 2005; Suzuki, Li, Sun, & Chiang, 2006). The *CesA* gene number in gymnosperms is similar to the one in angiosperms. For example, eight *CesA* genes were found in *Pinus radiata* (Krauskopf, Harris, & Putterill, 2005), 17 *CesA*s in *Cunninghamia lanceolata* (Huang et al., 2012), six in *P. taeda* (Neale et al., 2014), and nine *CesA*s in *G. montanum* (Wan et al., 2018), in comparison with 10 and 18 *CesA* genes in *Arabidopsis* and *Populus trichocarpa* genomes, respectively (Suzuki et al., 2006). Recently, in the *G. montanum* genome reference paper, it was suggested that large expansions in the *Csl*B/H subfamilies may explain the distinct growth characteristics in *Gnetum* when compared to other gymnosperms (Wan et al., 2018). It is interesting that *Csl*B/E/H/G that evolved from ancestral genes in ferns were lost in many gymnosperms, such as *P. abies*, *P. taeda*, *G. biloba*, and other species (Yin et al., 2014).

Cellulose and hemicellulose biosyntheses are regulated at the transcriptional level (Li, Bashline, Lei, & Gu, 2014). In angiosperms, for example, at least 13 out of 126 MYB transcription factors were reported to be involved in cellulose formation by regulating *CesA/Csl* gene expression directly or indirectly in *Arabidopsis* (Zhang, Nieminen, Serra, & Helariutta, 2014). However, in gymnosperms, only 13 *Picea glauca* and five *P. taeda MYB* genes were identified, suggesting a much lower number of MYB genes than in *Arabidopsis* and *Populus* (Bedon, Grima‐Pettenati, & Mackay, 2007). Some gymnosperm MYB genes, which have conserved functions (e.g., *PtMYB1* and *PtMYB4* in *P. taeda*), are expressed in the secondary xylem and involved in lignin biosynthesis as their homolog in *Arabidopsis* (Bedon et al., 2007; Patzlaff et al., 2003). Whether cellulose biosynthesis is regulated by MYB transcription factors is not clear in gymnosperms. However, the *CesA* genes' regulation network in gymnosperms might be less complex than in angiosperms. Cellulose biosynthesis is also affected by the content of lignin, another component of the plant cell wall (Endler & Persson, 2011). In *Populus*, artificial lignin biosynthesis inhibition is coupled with cellulose production and higher growth, suggesting cellulose synthase activity is restricted by substrate content (Hu et al., 1999). In *P. taeda*, spontaneous mutations in lignin biosynthesis (Songstad, Petolino, Voytas, & Reichert, 2017) also caused fast stem growth, suggesting cellulose synthase activity may be naturally regulated by lignin content in gymnosperms (Gill, Brown, & Neale, 2003).

### 4.2 Vascular NAC domain

The difference between water‐conducting xylem tissues (tracheids vs. vessels) is one of the main differences between gymnosperms and angiosperms (Wan et al., 2018). Tracheids, whose dual function is water transport and mechanical support, constitute the xylem tissue in gymnosperms. In angiosperms, xylem tissue is more complex and consists of vessels, fibers, and rays (Patten, Vassão, Wolcott, Davin, & Lewis, 2010 2010). In angiosperms, vessel cells are differentiated from cambium cells that undergo secondary cell wall biosynthesis and programmed cell death (Zhang et al., 2014). VASCULAR‐RELATED NAC‐DOMAIN6 (VND6) and VND7 are the key transcription switches on vessel element formation in *Arabidopsis* via activation of the transcription cascade involved in secondary cell wall biosynthesis and programmed cell death (Kubo et al., 2005; Ohashi‐Ito & Fukuda, 2014; Zhong, Lee, & Ye, 2010). There are seven *VND* genes encoded in the *Arabidopsis* genome, and *VND1*‐*5* was also recently reported to be involved in vessel element formation (Tan et al., 2018; Zhou, Zhong, & Ye, 2014). All of the seven *VND* genes in *Arabidopsis* were specifically expressed in vessels and had conserved downstream targets controlling vessel formation (Zhong, Lee, Zhou, McCarthy, & Ye, 2008; Zhou et al., 2014). It is interesting that the *P. abies* and *G. montanum* genomes only encode two and one *VND* ortholog genes, respectively, and both of them are homologous to *VND4*/*5*/*6* (Nystedt et al., 2013; Wan et al., 2018). There are two possible explanations for why vessels are absent in gymnosperms. In the first one, *VND1*–*3* and *VND7* may determine function in vessel formation (Wan et al., 2018), and at least, the dominant repression of VND7 showed a more severe phenotype than the dominant repression of VND6 (Kubo et al., 2005). In the second one, vessel formation requires VND gene expansion and their co‐expression (Nystedt et al., 2013). Although the seven VNDs in *Arabidopsis* had conserved expression patterns and downstream genes, the expression level in vessels of different organs and activation strength were different (Zhou et al., 2014), suggesting the seven VNDs might coordinately work to regulate vessel formation.

## 5 SECONDARY METABOLISM AND STRESS‐RELATED GENE FAMILIES

### 5.1 Abiotic stress—Dehydrins

Dehydrins are a group of proteins belonging to the late embryogenesis abundant (LEA) gene family that are highly hydrophilic and are commonly associated with acclimation to low temperature and other environmental stresses involving cellular dehydration in plants (Rorat, 2006). Dehydrins have been shown to be related to drought tolerance (Hu, Wang, Du, & Huang, 2010; Lopez, Banowetz, Peterson, & Kronstad, 2003; Suprunova et al., 2004) and low temperature acclimation (Danyluk et al., 1998; Gao & Lan, 2016; Strimbeck, Schaberg, Fossdal, Schröder, & Kjellsen, 2015) in several species. One study on *Picea obovata* showed a dehydrin accumulated to ~16× its initial level during acclimation from moderate to extreme low temperature tolerance (Kjellsen, Shiryaeva, Schröder, & Strimbeck, 2010). Multiple studies demonstrate a similar relationship in other taxonomic groups, suggesting a strong association between low temperature acclimation and accumulation of dehydrins across taxonomic groups (Arora & Wisniewski, 1994; Kontunen‐Soppela & Laine, 2001; Liu et al., 2004; Renaut, Hoffmann, & Hausman, 2005; Rinne, Welling, & Kaikuranta, 1998). Furthermore, significant differentiation in allelic frequency has been observed at three dehydrin‐associated loci between populations of *P. sylvestris* L. showing divergence for cold tolerance (Wachowiak, Balk, & Savolainen, 2009). However, their data suggest that nucleotide polymorphism in most *P. sylvestris* dehydrins cannot be directly related to adaptive variation in cold tolerance (Wachowiak et al., 2009).

The specific mode of action of dehydrins is unclear, but some studies suggest that dehydrins stabilize membranes and macromolecules in conditions of low water availability (Hanin et al., 2011). The size of the dehydrin gene family is highly variable ranging from two members in *Amborella* to more than 12 in *Malus domesticus* in angiosperms. Gymnosperms are less studied, but within Pinaceae, the dehydrin family appears to be much larger relative to angiosperms, with a total of 53 having been identified in *P. glauca* (Stival Sena, Giguère, Rigault, Bousquet, & Mackay, 2018). Subfunctionalization is thought to be the primary driver for the increased diversity of dehydrins in conifers over angiosperms (Stival Sena et al., 2018). In contrast, extant species of *Gnetum* have reduced numbers of *LEA* genes (and dehydrins) when compared to other gymnosperms (Wan et al., 2018). *Gnetum* also differs from other gymnosperms in that it only exists in warm, mesic habitats (Wan et al., 2018), lending more evidence to the role dehydrins play in adaptation to water stress.

### 5.2 Defense systems

Conifer defenses against pests and pathogens involve many different gene families, and many of them have been well‐studied in terms of their occurrence within the genome (Warren et al., 2015; Zhou et al., 2019). Some of these include biosynthetic enzymes like the ones acting in oxygenation, phenoxy radical coupling or regio‐ or stereo‐selective reactions resulting in an immense diversity of defense compounds (for induced defenses see: Keeling & Bohlmann, 2006; Kovalchuk et al., 2015; Oliva et al., 2015; Ralph, Yueh, et al., 2006; Ralph, Park, Bohlmann, & Mansfield, 2006; Ralph, Jancsik, & Bohlmann, 2007; Visser, Wegrzyn, Myburg, & Naidoo, 2018; for constitutive defenses see: Keeling & Bohlmann, 2006; Ralph et al., 2007; Porth et al., 2011; Porth et al., 2012; Porth et al., 2018). Regulatory genes include the Sg4C R2R3‐MYB transcription factor that exhibits a significant gene family expansion in conifers (Bedon et al., 2010). Moreover, crucial biosynthetic genes for pest resistance (e.g., 3CAR; CYP720B4) feature high content of repetitive sequence regions and transposable elements, suggesting that diversification of the conifer TPS and P450 gene families may have been achieved by DNA transposon‐mediated translocation mechanisms (Hamberger et al., 2009). Another important feature of conifer TPSs is their high potential for functional plasticity such that few changes in amino acids can create new potent defense molecules (Keeling, Weisshaar, Lin, & Bohlmann, 2008).

Because plants have a long evolutionary history of interaction with herbivores, hosts have acquired coevolved defenses (Futuyma & Agrawal, 2009). A special case is the gymnosperm *G. biloba*, which is largely herbivore‐free. Ginkgo's foliage produces ginkgolides, a class of terpene trilactones known as a potent antifeeding defense (Mohanta et al., 2012; Pan, Ren, Chen, Feng, & Luo, 2016). In general, the most effective host tree defenses exist against local pests and pathogens, while host defenses weaken under relaxed or absent pathogen pressure. This is a recurrent problem with introduced foreign pest and pathogens, but also with native pests and pathogens expanding their natural ranges. As climate warms, these native species may expand their ranges northwards or to higher altitudes, where they may encounter “naïve” hosts. Moreover, native species may change their metabolism to support a more aggressive behavior, leading to unprecedented population growth and range expansions, and threatening local and new host trees in a pest's newly invaded habitat. A widely publicized example of current range expansion is the mountain pine beetle (*Dendroctonus ponderosae* Hopkins). This pest epidemic in western North America is now threatening the boreal forest (Cullingham et al., 2011).

Trees have developed different lines of defense that are more or less effective, and also alternative strategies such as tolerance. Anatomical and the associated chemical defenses in conifer bark have been described (Franceschi, Krokene, Christiansen, & Krekling, 2005). Strength and rapidity of traumatic resinosis (direct defense) has often been associated with resistance. The physical structures studied in most detail are the parenchyma cells (locations of synthesis and storage of polyphenols), and the resin ducts (synthesis and storage of terpenes) that are located in the secondary phloem and the cambium. The traumatic resin canals are formed in the secondary xylem as a way of active defense. Upon attack, reallocation of resources from primary processes to active defense, or the mobilization of the resources for host tolerance, takes place. Indirect tree defense responses that involve the attraction of predators or herbivore parasitoids have also been documented. Moreover, trade‐offs involving defense strategies involve display of chemical defenses, or rely on tolerance (Futuyma & Agrawal, 2009). In a recent study on the genomics of host defenses against the spruce shoot weevil (*Pissodes strobi* Peck), Porth et al. (2018) concluded that well‐established terpenoid‐related spruce defenses and tolerance to this herbivore might be mutually exclusive.

It has been postulated that drought‐stressed conifers whose metabolism is diverted from growth to secondary compounds can rely more on constitutive, preformed defenses (Turtola, Manninen, Rikala, & Kainulainen, 2003). Also, it is well known that fast growing individuals are biased toward induced defenses (Steppuhn & Baldwin, 2008). Therefore, trade‐offs between already established and induced defenses can be expected. These dynamics under different environmental conditions need to be better studied in the future, while current genomic studies usually represent a snap‐shot situation aiming to identify few highly upregulated candidate genes from well‐annotated conifer defense metabolic pathways such as the phenylpropanoid and methylerythritol phosphate/mevalonate (Hall, Yuen, et al., 2013a; Keeling et al., 2011; Porth et al., 2011; Shalev et al., 2018; Warren et al., 2015; Zhou et al., 2019). In addition, the genetic networks between defenses in conifers and their reproductive development seem to be intricate. With few exceptions, this important relationship has been largely ignored in conifer defense studies, mainly because the conifer reproductive genes (many are also gene family members) were under‐studied; thus, their exact functioning remains elusive (see section on Reproductive Biology). In any case, it is known that certain signaling pathways (jasmonate, ethylene, auxin, gibberellin) required for developmental processes (such as those important in reproduction) can be co‐opted for biotic stress responses (e.g., Du et al., 2017; Oliva et al., 2015; Thaler, Farag, Paré, & Dicke, 2002; Zi, Mafu, & Peters, 2014). Alternatively, these signaling pathways may evolve into new specialized pathways such as the conifer defensive resin production co‐opted from gibberellin production (Zi et al., 2014).

Given the current knowledge about defensive gene family expansion in gymnosperms (Porth et al., 2011, 2012; Warren et al., 2015; Zhou et al., 2019), the challenge remains to identify the most potent defensive metabolites against herbivory or disease produced in these pathways. Here, we show examples of natural host defense compounds with proven dramatic negative impacts on pest development in *Picea*. Delvas, Bauce, Labbé, Ollevier, and Bélanger (2011) identified acetophenones that act against the budworm *Choristoneura fumiferana*. Robert et al., 2010 showed 3‐carene and dehydroabietic acid acting against the weevil *P. strobi*. Moreover, (+) catechin was found effective against fungal pathogenicity in the form of *Heterobasidion parviporum* (Nemesio Gorriz et al., 2016). The work by Liu and Ekramoddoullah (2007, 2011) showed CC‐NBS‐LRR and TIR‐NBS‐LRR expression conveying resistance against white pine blister rust (caused by *Cronartium ribicola*) in western white pine.

For resistance breeding purposes, knowledge about defense metabolites' heritability (i.e., the extent of their genetic control) in the breeding population is required. The work by Méndez‐Espinoza et al. (2018) on acetophenones' genetic parameters remains the only work on this aspect to date. In the case of genetic marker‐assisted resistance breeding, it is important to ascertain the underlying genetic regulation for compounds of interest. This information is only available for a few metabolites. For example, Roach, Hall, Zerbe, and Bohlmann (2014) identified the related 3‐carene synthase, 2‐sabinene TPSs. Hamberger, Ohnishi, Hamberger, Seguin, and Bohlmann (2011) found the specific cytochrome P450 of the CYP720B family implicated in dehydroabietic acid synthesis. Mageroy et al. (2015) found β‐glucosidase, the biosynthetic gene for acetophenones. Finally, Nemesio Gorriz et al. (2016) isolated a leucoanthocyanidin reductase for (+) catechin generation. Other studies have focused on identifying the biosynthesis locations for terpenoids (Abbott, Hall, Hamberger, & Bohlmann, 2010; Zulak & Bohlmann, 2010) and phenolic (Li et al., 2012) compounds to better target effective tree defenses in the future.

### 5.3 A case study of functional pleiotropy with defense: the PDR ABC transporter family

*Pleiotropic drug resistance* (*PDR*) genes belong to a fungi and plant‐specific gene family within the *ATP Binding Cassette (ABC)* gene superfamily (Crouzet, Trombik, Fraysse, & Boutry, 2006; Higgins, 1992; Lamping et al., 2010). The *PDR* gene family was named following the observation that members of its family confer resistance to various drugs; however, *PDR* genes are also involved in the transport of substrates not related to cell detoxification (Ito & Gray, 2006; Nuruzzaman, Zhang, Cao, & Luo, 2014; Pierman et al., 2017; Sasse et al., 2016). Three recent and completely independent studies on two spruces (*P. glauca*; *P. glauca *×* engelmannii*) and *P. taeda* are suggesting specific *PDR* genes as important key players in defense mechanisms against different herbivores (Mageroy et al., 2015; Porth et al., 2018) and pathogens (De la Torre et al., 2018). For example, research on spruce budworm (*C. fumiferana*) resistance identified gene *WS0269_K02* with high statistical support for its expression upregulation in budworm resistant versus nonresistant white spruces (Mageroy et al., 2015; information drawn from their Table S1). The same *WS0269_K02* gene was found in spruce shoot weevil (*P. strobi*) resistance (Porth et al., 2018, Figure 2). In pine, a closely related gene family member was identified for pitch canker disease (*Fusarium circinatum*) resistance (De la Torre et al., 2018). Because these genes' expressions were also correlated with drought resistance (De la Torre et al., 2018) and growth rate (Porth et al., 2018), genetic pleiotropic functioning of conifer *PDR* genes could be implied. Drought resistance and growth might share a genetic relationship to a certain extent, as trees impaired in drought tolerance and succumbing to drought stress are expected to show decreased growth (Salmon et al., 2019). It has further been postulated that drought‐stressed conifers rely more on constitutive than on induced defenses (Turtola et al., 2003).

**Figure 2:**
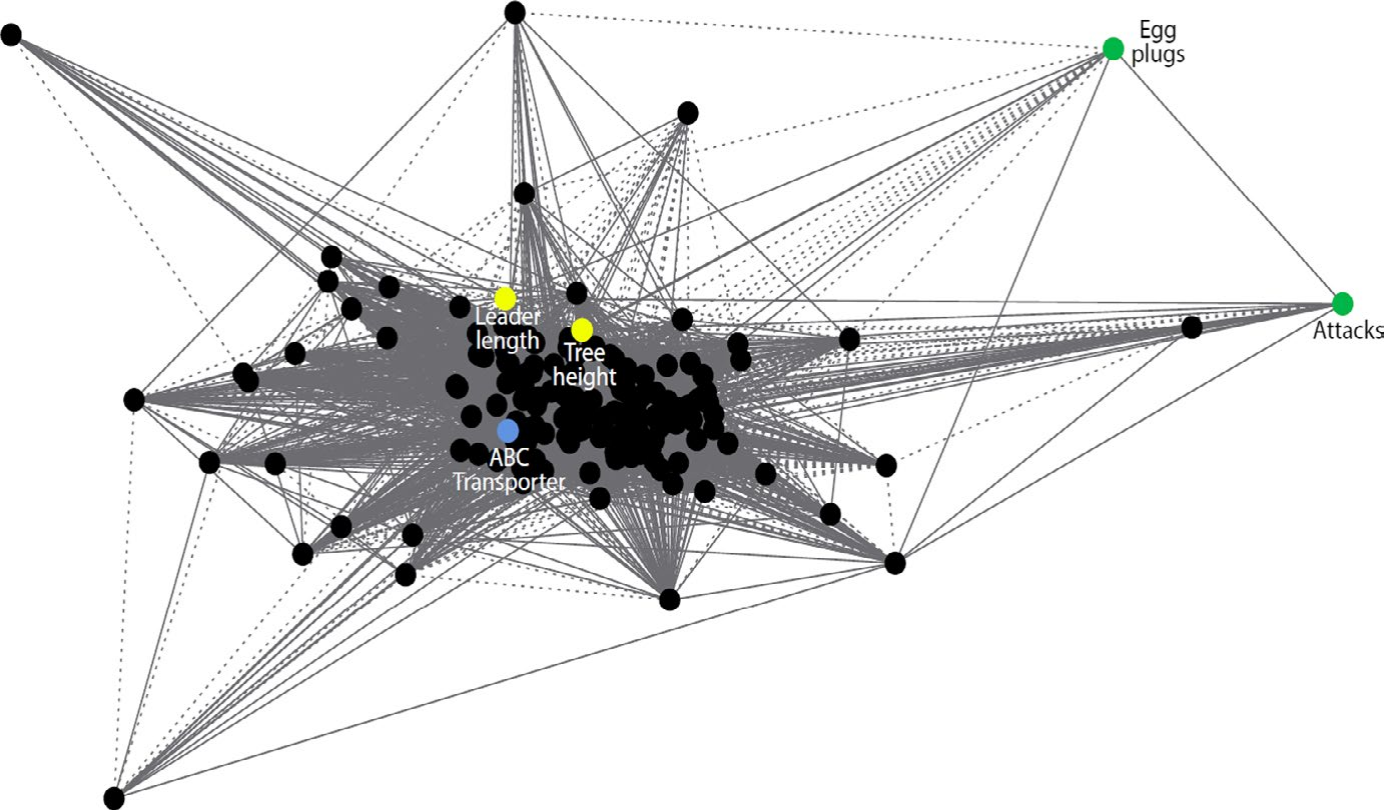
The white spruce PDR gene family member *WS0269_K02* identified as a core gene. Spruce PDR gene (ABC transporter, blue dot) identified as “core gene” (Porth et al., 2018) in the gene regulatory network with growth (yellow dots) or defense phenotypes (against the stem‐boring spruce shoot weevil *Pissodes strobi*; green dots)

**Figure 3:**
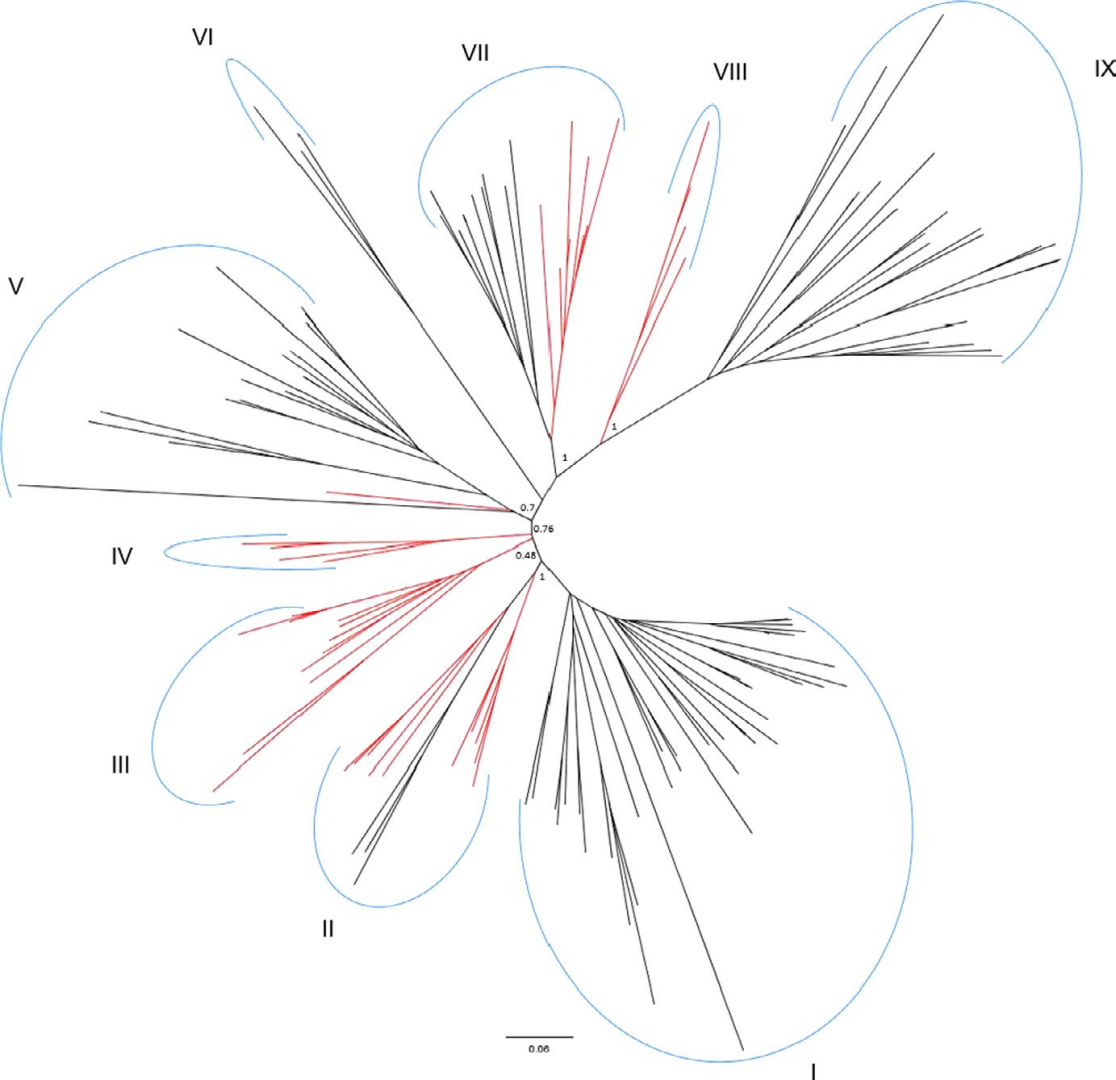
Phylogenetic analysis of vascular plant PDR protein sequences obtained by maximum likelihood. Protein sequences were arbitrarily grouped into nine clusters (I–IX) based on close sequence similarity. Red branches are leading to PDR sequences belonging to gymnosperm sequences. Sequence names have been pruned from the tree for the sake of clarity. Sequence names belonging to each cluster can be found in Table S1. Node support from 1,000 replicates is indicated for the basal nodes defining the nine putative PDR sequence clusters. For further details, see Appendix S2

Our study found that the size of the PDR family in gymnosperms is smaller compared to angiosperms. This may indicate that gymnosperm species require less PDR transporters than angiosperms to cope with their environment. The identified conifer *PDR* gene sequences were further mapped to the *PDR* genes' phylogenetic tree for improved annotations (Figure 3). In the case of the white spruce gene (identified by Mageroy et al., 2015 and Porth et al., 2018), *WS0269_K02* mapped to cluster IV, a gymnosperm‐specific clade, and it was found to be putatively identical to the *P. abies* gene *Pab_MA_17319g0010*, Table S1. In the case of the *P. taeda* gene (c3387/f1p0/2274 identified in De la Torre et al., 2018), its transcript mapped to cluster II on the phylogenetic tree (sister to a group of the two conifer sequences Pab_MA_10427561g0010 and Pta_04241). Two hypotheses can be proposed to explain the diversification of the *PDR* gene family. First, *PDR* genes might have diversified by acquiring new physiological roles (neofunctionalization). Different plant species produce different metabolites, and diversification is expected in enzymes involved in the transport of these metabolites (Yazaki, 2006). Secondly, the differential expression of *PDR* genes in different tissues or during different developmental stages might have promoted their diversification (subfunctionalization). To fully grasp the evolution of the *PDR* gene family, more *PDR* gene sequences from additional species across the plant kingdom are needed to better resolve *PDR* gene evolution and relationships (this was beyond the scope of the present study).

## 6 NONCODING AND SMALL RNAS

Noncoding RNAs are a class of RNAs not involved in protein coding, but with very important functions as regulators in plant life cycle, response to the environment, and phenotypic plasticity (Borges & Martienssen, 2015; Shin & Shin, 2016). Noncoding RNAs can be divided into two categories, long noncoding RNAs (>200 nucleotides, nt) and small noncoding RNAs (sRNA) (20–24 nt) (Arikit, Zhai, & Meyers, 2013). Differences in sRNA size distribution can be observed between gymnosperms and angiosperms. The 21‐nt sRNAs are dominant in gymnosperms such as *P. abies* (Nystedt et al., 2013), *Pinus contorta* (Morin et al., 2008), *Larix leptolepis* (Zhang et al., 2013), and *P. tabuliformis* (Niu et al., 2015), whereas 24‐nt sRNAs represent the majority in angiosperms (Li et al., 2017; Morin et al., 2008). For a long time, 24‐nt sRNAs were thought to be absent from gymnosperms, and now, we know they occur at low frequencies and are mainly restricted to reproductive tissues (Niu et al., 2015; Nystedt et al., 2013; Zhang et al., 2013). Therefore, the presence of 24‐nt sRNAs may be important in the regulation of reproduction in gymnosperms (Niu et al., 2015). Because 21‐nt sRNAs are involved in target gene silencing or protein translation inhibition and 24‐nt sRNAs are functional on chromatin remodeling (Borges & Martienssen, 2015), it seems that sRNAs may play different regulatory roles in gymnosperm and angiosperm development, respectively.

In plants, the biogenesis of sRNA is dependent on dsRNA precursor cleavage mediated by DICER ribonucleases (DCL; Van Ex, Jacob, & Martienssen, 2011). DCL1 and DCL4 generate 21‐nt sRNAs, while DCL3 generates 24‐nt sRNAs. It is interesting that short variants of DCL1 were characterized in *P. lambertiana* (Gonzalez‐Ibeas et al., 2016) and in the bryophyte *Physcomitrella patens* (Coruh, Shahid, & Axtell, 2014). This finding suggests that functional truncated DCL1 might be the reason why 21‐nt sRNAs are dominant in gymnosperms, although the relationship between conifer‐specific 21‐nt sRNA and short DCL1 is unclear (Gonzalez‐Ibeas et al., 2016). A conifer‐specific set of DCL1 proteins was found in *P. glauca, P. abies,* and *P. lambertiana* (Dolgosheina et al., 2008; Gonzalez‐Ibeas et al., 2016). DCL3, which is involved in 24‐nt sRNAs biogenesis, was characterized through *P. lambertiana* transcripts, primarily expressed in reproductive tissues (Gonzalez‐Ibeas et al., 2016). Truncated DCL3 was also discovered in *P. glauca,* and its expression level upregulated in seed development indicated that the DCL3 variant and its expression level are responsible for 24‐nt sRNA generation in *P. glauca* (Liu & El‐Kassaby, 2017). The discovery of variant DCL partly explained the different sRNA size distribution between gymnosperms and angiosperm, although further confirmation is needed. The 24‐nt sRNAs direct DNA methylation and affect histone modification which are related to chromatin condensation and silencing of transposable elements (Leitch & Leitch, 2012). The different silencing mechanisms were correlated with differences in genome sizes of angiosperms and gymnosperms (Dolgosheina et al., 2008; Leitch & Leitch, 2012).

## 7 APPLICATIONS OF THE STUDY OF GYMNOSPERM GENE FAMILIES

Plant defense molecules are highly complex traits with nutritional value, flavor, and use in traditional medicine (Hamberger & Bak, 2013). Genes encoding natural product pathways often group together in biosynthetic gene clusters (Nützmann, Huang, & Osbourn, 2016). Some of the genes reviewed in this study are newly studied members of gene families that hold great potential for biotechnological applications related to commercial and pharmacological value. The plant‐based dirigents (Pickel & Schaller, 2013), cytochrome P450s (Hamberger & Bak, 2013; Renault, Bassard, Hamberger, & Werck‐Reichhart, 2014), and terpenoids (Bohlmann & Keeling, 2008; Singh & Sharma, 2015) have been recognized to hold potential for biotechnology. Because of the large metabolic diversity arising from the multitude of biochemical reactions for these gene families' members (P450s: Hamberger & Bak, 2013; TPSs: Boutanaev et al., 2015; Chen et al., 2011; Chen et al., 2018; conifer TPSs: Hall, Zerbe, et al., 2013b; Zerbe et al., 2012; dirigents: Pickel & Schaller, 2013), new plant natural product pathways are likely to be discovered and functionally described. Perhaps the most famous compound of terpenoid origin for human uses is taxol, a potent anticancer drug (Wani, Taylor, Wall, Coggon, & McPhail, 1971) and whose biosynthesis in *Taxus* spp. has been elucidated (Croteau, Ketchum, Long, Kaspera, & Wildung, 2006). Pseudolaric acid B, a diterpene acid (originating from *Pseudolarix kae*mpferi bark), is another well‐known drug that reduces tumor growth, in particular for melanoma (Gong, Wang, Tashiro, Onodera, & Ikejima, 2005). With ~50,000 different molecules identified in extant plants, terpenoids are structurally and functionally the most diverse plant metabolic group. They are of substantial commercial and pharmacological value as essential oils, fragrances, colorants, drugs, coatings, and speciality plastics (Sainz et al., 2016; Vranová, Coman, & Gruissem, 2012). For example, conifer terpenoid oleoresins are used by the naval stores industry to create rosin and turpentine used as adhesives, inks, solvents, and resins. Although their production was reduced due to the increase of less expensive petroleum‐derived substitutes, terpenoid oleoresin might come back as an important source of “green” biofuels and bioproducts (Turner, Parrish, Zager, Fischedick, & Lange, 2018). Environmental and developmental factors affect the terpenoid pathway flux; understanding the complexity of the terpenoid pathway network in plants and its regulation remains a major challenge in terpenoid research but will facilitate future molecular breeding of agronomically useful traits (Vranová et al., 2012).

Some members of conifer gene families (such as the *PDR* gene family) can also be exploited for their potential to improve conifer tree growth on marginal or disturbed soils, thus providing an improved detoxification potential to employ conifers (i.e., spruces) in phytoremediation applications. In addition, functional characterization of *PDR* genes is required before biotechnology applications can be performed on the *PDR* gene family, particularly for long‐lived trees (Lefevre, Baijot, & Boutry, 2015). Because PDRs have been shown to act in a variety of plant organs, above ground (foliage and reproductive structures) and below ground (in roots; Crouzet et al., 2006), one of the most intriguing applications besides phytoremediation is the PDR's potential in conferring improved resistance to biotic stressors (De la Torre et al., 2018; Mageroy et al., 2015; Porth et al., 2018). Also, a better knowledge of the genes and gene families conferring phenotypic variation is the first step to create plantations with improved varieties through marker‐assisted breeding, genomic selection, or genetic modifications (CRISPR). For species with ecological importance, the identification of genes families involved in abiotic and biotic stress may contribute to identify species that are candidates to ecological restoration, or that may present increased potential to adapt to specific or changing climatic conditions.

## 8 CONCLUSIONS

In this paper, we aim to understand how genes and gene families have contributed to the evolution of major functional differences in gymnosperms in comparison with its sister plant clade of flowering plants. Recently developed new reference genomes, transcriptomes, and genome‐wide resources in gymnosperms have enabled large‐scale comparisons of functional divergence within gymnosperms, and between angiosperms and gymnosperms. Information about the genomic architecture underlying phenotypic variation is key for any applied breeding and management of commercially important gymnosperm species. With the development of new genomic tools and analytical software, future approaches will include a higher contiguity of reference genomes; completion of structural and functional annotation of reference genomes; increase in the number and density of physical, linkage, or genetic maps; whole‐genome re‐sequencing of populations for GWAS studies; and genetic improvements through biotechnology.

## DATA ARCHIVING STATEMENT

This manuscript has no associated data for data archiving.
