# Supplementary material for: Functional and morphological evolution in gymnosperms: A portrait of implicated gene families
Source: Evol Appl. 2019 Jul 21;13(1):210–27. doi: 10.1111/eva.12839 (PMC6935586; doi:10.1111/eva.12839)
Supplement: Supplementary file 2 [file EVA-13-210-s002.docx]

**Evolutionary Applications – Appendix 2**

*PDR* genes’ amino acid sequences from three different gymnosperms, Norway spruce (*Picea abies)*, loblolly pine (*Pinus taeda*) and Ginkgo (*Ginkgo biloba*) were retrieved using *BLAST+* (Camacho et al*.,* 2009)*.* In addition, we retrieved *PDR* sequences from two angiosperm trees, the black cottonwood (*Populus trichocarpa)* and pedunculate oak (*Quercus robur*) for comparison. We blasted *Arabidopsis thaliana* PDR proteins against protein databases from these five species. Only sequences longer than 800 amino acids were retained in the alignment to avoid including non-PDR ABC proteins or truncated protein sequences. Other PDR protein sequences from six angiosperm model species were download from NCBI. Alignment of full-length protein sequences was performed using the MUSCLE algorithm (Edgar, 2004). A phylogenetic tree was inferred by maximum likelihood using aligned PDR proteins in RAxML (Stamatakis, 2014).

Using the 15 PDR amino acid sequences of *A. thaliana* as a query, we identified 12 PDR proteins in *Picea abies,* 17 in *Gingko biloba,* 18 in *Pinus taeda*, 25 in *Quercus robur*, and 27 in *Populus trichocarpa.* The phylogenetic tree resulting from the protein alignment included 11 vascular plant species and 137 PDR proteins (**Fig. 3**). We arbitrarily grouped amino acid sequences into clusters based on their close sequence similarity to better understand gene evolution in this family. Support values for basal nodes defining each cluster are relatively high (>0.7) except for one node. Gymnosperms represent an older taxon compared to angiosperms, thus we expect gymnosperm *PDR* sequences to branch at the base of angiosperm *PDR* sequences. This is observed only in clusters II, V and VII. Some clusters include only *PDR* sequences belonging to angiosperm species (cluster I, VI and IX) while some other clusters include only sequences from gymnosperm species (cluster III, IV and VIII). PDR protein numbers ranged from 12 to 18 in gymnosperm species and ranged from 15 to 27 in angiosperm species for which all known *PDR* sequences were used (*i.e. Arabidopsis thaliana*, *Oryza sativa, Q. robur* and *P. trichocarpa*).

Here, we assessed the diversity of the *PDR* gene family in three gymnosperm species and compared them to other angiosperm species in a phylogenetic context. The grouping of *PDR* sequences in 9 clusters allowed us to better interpret the evolution of this gene family. One basal node of the phylogenetic tree, however, has a relatively low support indicating that the tree topology found in this study may change if using a more complete dataset. However, given the small size of our dataset, this result may not represent a global trend between gymnosperms and angiosperms. We observed that some amino acid sequence clusters included both angiosperm and gymnosperm sequences. This is expected as PDR protein diversity in plants happened after the separation of plants and fungi (Crouzet et al., 2006). Therefore, some *PDR* genes should present a close similarity if they evolved under purifying selection or under similar evolutionary pressures. Other clusters, presenting only gymnosperm or angiosperm sequences indicate that new *PDR* genes appeared to form new clusters or inversely that genes disappeared from existing clusters.

**References**

Crouzet, J., Trombik, T., Fraysse, A. S., & Boutry, M. (2006). Organization and function of the plant pleiotropic drug resistance ABC transporter family. *FEBS Letters*, *580*(4), 1123–1130. Retrieved from http://www.ncbi.nlm.nih.gov/pubmed/16506311

Edgar, R. C. (2004). MUSCLE: multiple sequence alignment with high accuracy and high throughput. *Nucleic Acids Research*, *32*(5), 1792–1797. https://doi.org/10.1093/nar/gkh340

Stamatakis, A. (2014). RAxML version 8: a tool for phylogenetic analysis and post-analysis of large phylogenies. Bioinformatics, 30(9), 1312–1313. https://doi.org/10.1093/bioinformatics/btu033
